# Neonatal care practice and factors affecting in Southwest Ethiopia: a mixed methods study

**DOI:** 10.1186/s12914-015-0050-2

**Published:** 2015-07-20

**Authors:** Gurmesa Tura, Mesganaw Fantahun, Alemayehu worku

**Affiliations:** Department of Reproductive Health and Health Service Management, School of Public Health, Addis Ababa University, P.O.Box: 378, Addis Ababa, Ethiopia; Department of Epidemiology and Biostatistics, School of Public Health, Addis Ababa University, Addis Ababa, Ethiopia

**Keywords:** *Neonatal care practice*, *Factors affecting neonatal care*, *Southwest Ethiopia*, *Multilevel linear regression*, *Mixed methods study*

## Abstract

**Background:**

A significant proportion of neonatal mortality can be prevented by the provision of the minimum neonatal care package. However, about 3 million neonates die each year globally because of lack of appropriate care. This situation is the worst in Ethiopia. Thus, the objective of this study was to determine the status of neonatal care and identify factors affecting.

**Methods:**

A mixed methods study involving both quantitative and qualitative methods was conducted from September 2012-December 2013 in Southwest Ethiopia. Randomly selected sample of 3463 mothers were interviewed to collect the quantitative data. Twelve in-depth interviews with purposively selected key informants and six focus-group discussions with purposively selected mothers were conducted for the qualitative data. Mixed-effects multilevel linear regression model was used to identify predictors of neonatal care practice by using STATA 13. Audio recording, transcription and thematic content analysis was done for the qualitative data.

**Results:**

The overall status of neonatal care practice was 59.5 % (95 % CI: 57.6 %, 61.3 %). Of the respondents, 53.8 % received tetanus toxoid, 23.8 % planed for birth, 41.9 % received at least one antenatal care and 43.0 % received adequate information during pregnancy. Only, 17.5 % received skilled care at birth and 95.0 % received social support. Of the neonates, 96.5 % received appropriate thermal care, 86.5 % received clean cord care, 64.1 % initiated breast-feeding within one hour, 91.5 % were on exclusive breast-feeding, 56.5 % received appropriate bathing and 8.1 % received vaccination on date of birth. Place of residence, maternal education, husband’s occupation, wealth quintiles, birth order and inter-birth interval were identified as predictors of neonatal care practice.

**Conclusions:**

The status of neonatal care practice was low in the study area. Skilled care at birth and receiving vaccination on date of birth were the worst practices. Factors affecting neonatal care existed both at cluster level and at the individual level and included socio demographic, economic and obstetric factors. Appropriate birth spacing, birth limiting and behaviour change communications on the importance of neonatal care are recommended.

## Background

Worldwide, about 3 million neonates die each year because of lack of appropriate care [1]. The biggest burden (98 %) of this neonatal death is shouldered by the low and middle-income countries. Sub-Saharan Africa is among the regions showing the least progress in reducing neonatal mortality [1]. Most of these deaths are caused by infectious diseases, pregnancy-related complications and delivery-related complications, including intra-partum asphyxia, birth-trauma and premature birth, all of which can easily be prevented by providing the appropriate package of neonatal care [2, 3].

The global health actors, including researchers, policy makers and program implementers have been searching for new knowledge and technologies for child survival for many years. However, the issue of neonatal survival remained unfinished agenda and is among the unachieved millennium development goal targets identified for the post-2015 priorities [4, 5].

Ethiopia is among the low-income countries with the highest rate of neonatal mortality (37 deaths per 1000 live births) and showing very slow progress. Though the country is among the fast progressing countries in reducing under-five mortality and achieving the millennium development goal 4 (MDG_4_), the challenge of neonatal mortality is still a quandary [6–9].

Evidences suggest that a significant proportion of this neonatal mortality can be prevented by inexpensive, simple practices and interventions along the continuum of care starting from pre-pregnancy, during pregnancy, delivery and postnatal period [6–8, 10–12]. As part of neonatal survival strategies, The Lancet Neonatal Survival Steering Team identified evidence-based cost-effective interventions and published in 2005 [13]. Following this, the World Health Organization (WHO) developed a minimum neonatal care package to be given during pregnancy, labor and childbirth, the immediate postpartum period and the first 28 days of life [14]. This minimum neonatal care package includes tetanus toxoid (TT) immunization, birth preparedness and complication readiness (BPCR), antenatal care (ANC) visit and adequate information on neonatal care during pregnancy; skilled care and social support during labor and delivery and immediate thermal care, clean and safe cord care, timely and exclusive breast feeding, appropriate bathing time and immunization on date of birth during the post partum period (15).

Almost all countries, including Ethiopia, have adopted this strategy and have been implementing for nearly a decade. However, newborn care often receives less-than-optimum attention. Besides, newborn care is strongly influenced by women’s social and health status and by home care practices for mother and newborn as well as by maternal and newborn care services. Traditional care practices during delivery and immediately for newborn at home and in the community inevitably affect the health of the neonates. However, the major challenge is lack of adequate data about the local practices at the community level for program improvement [15–18].

Thus, it is crucial to have up-to-date information on the existing neonatal care practices and affecting factors to design more successful interventions. To the investigators’ knowledge, the status of provision of the minimum neonatal care package and affecting factors have not been well assessed in the study area. Therefore, the purpose of this study was to determine the status of neonatal care practice and identify factors affecting by conducting community based mixed methods study in Jimma Zone Southwest Ethiopia.

## Methods

### Study design and setting

This community-based prospective follow up study, employing both quantitative and qualitative data collection methods, was conducted in Jimma Zone, Southwest Ethiopia from September 2012 to December 2013. Jimma Zone is one of the 17 Zones of the Oromia Regional State of Ethiopia. Administratively, the Zone is sub-divided in to 17 rural districts called ‘*Woredas*’ and two town administrations. According to the 2007 national population and housing census, the Zone has a total population of about 2.6 million, of whom 88.7 % are rural residents [19, 20].

### Population, sample size and sampling methods

Mothers who had given birth 28 days before the survey were the study populations for the quantitative method. The minimum required sample size for this study was determined by using Epi-Info V.3.5.1 based on the following assumptions. The status of neonatal care practice as determined by the mean score of composite variable (indices) was assumed to be 29 % (*p* = 0.29) (Gashaw A. Assessment of New Born Care Practices During the first week of life Among mothers in Addis Ababa, Ethiopia (Unpublished)). The allowed margin of error to be 3 % (d = 0.03) with 95 % level of confidence. In addition, as multistage-clustered sampling method was used, a design effect of 2 was considered. Finally, 10 % was added for non-responses and missed-to-follow up and the final sample size became 1934 mothers. This study was part of a bigger longitudinal study in which 3463 mothers were followed up. Therefore, to increase the precision of the estimates and power of the study, we included all the 3463 mothers in this study.

A multistage-clustered sampling technique was used to identify the study participants. Initially, the Zone was stratified as town administration and rural districts called '*Woredas*'. Then, 5 districts were selected by simple random sampling from the 17 districts. At the second stage, 9 rural ‘*Kebeles*’ and 2 urban ‘*Kebeles*’ were selected from each selected district randomly. Jimma town administration and Agaro town administration have 13 and 5 ‘*kebeles*’, respectively and all were included purposefully. With this, in total, 73 clusters (*'Kebeles'*) were included in the study from which 3682 pregnant women were enumerated and enrolled to the study at the baseline. All the enrolled pregnant women were followed till 28 days postpartum period and neonatal care practice was assessed at the end of neonatal period.

To have in-depth understanding of neonatal care practices and contributing factors, 12 in-depth interviews (IDIs) and 6 Focus Group Discussions (FGDs) were conducted. The IDIs involved 4 service providers, 4 traditional birth attendants (TBAs) and 4 Health Extension Workers (HEWs) all of whom were selected purposively based on their close relation with mothers and neonates and assumed to be rich sources of information on the topic of the study. The FGDs involved purposively selected 8-10 mothers having post neonatal infants (1-6 months) each. The number of IDIs and FGDs were determined based on level of saturation of the required information.

### Instruments and measurements

The data were collected by using pre-tested interviewer-administered structured questionnaires, which were adapted from related literatures. The indicators for the wealth index were adapted from Ethiopian Demographic and Health Survey (EDHS) [8]. Indicators for neonatal care practice were adapted from the World Health Organization (WHO) minimum neonatal care package [14]. The questionnaire was prepared in English, then translated to local languages ‘*Afan Oromoo’* and Amharic and used to collect the data.

The dependent variable for this study was neonatal care practice, which was a composite score (index) created from 12 items and treated as continuous variable. By taking ‘*Kebeles*’ as clusters, the independent variables were divided into two levels. Level-2 (higher level variables) included community or cluster-level variables such as place of residence, access to health centres and access to hospitals. Level-1 (lower- level variables) included individual and household characteristics such as: socio-demography, wealth quintiles and maternal obstetric factors. The detail description of each variable is given below (Tables 1 and 2).

**Table 1:** Description of variables and measurement for the study, Jimma Zone, Southwest Ethiopia, September 2012-December 2013

Variables	Descriptions	Measurements
Cluster (‘Kebele’)	The smallest administrative unit having about 5000 population on the average.	73 ‘Kebeles’ (clusters) identified by multistage sampling method were included in the study
Dependent variable		
Neonatal care practice	The minimum neonatal care package adapted from WHO having 12 items were used to create composite index (score) by using PCA.	The 12 items were measured in “Yes” or ‘No’ responses. Yes was given a value of ‘1’ and No was given ‘0’ to covert the categorical variables in to dummy variables. PCA was done to create composite index (score) which was a continuous variable.
The 12 items used to create neonatal care score (index)		
1. Tetanus toxoid (TT) during Pregnancy	Vaccination given for mothers during ANC visit to prevent the mother and her child against Tetanus	Those who received at least 1 dose of TT were coded as ‘Yes =1’ and otherwise ‘No = 0’
2. Birth preparedness and complication readiness (BPCR)	A package of interventions composed of 5 variables (Planed to save money, planed to arrange transport, identified place of delivery, identified skilled attendant and identified blood donor)	Composite variable was computed by adding the five responses. Women who scored 3 or more ‘Yes’ responses were categorized as ‘prepared = 1’ and otherwise ‘not prepared = 0’
3. Antenatal care (ANC)	Having health facility visit for pregnancy check up by skilled attendants during pregnancy	Those who had at least one ANC visit were labeled as "Yes = 1" and otherwise ‘No = 0’
4. Information on neonatal care	Included 4questions: Information on self care, breast feeding, neonatal feeding and vaccination.	Mothers who received the 4 information were labeled as ‘Received adequate information = 1’ and otherwise ‘No adequate information = 0’
5. Skilled care at birth	The place where the neonate was born and the person who assisted the mother during delivery	Deliveries conducted in health facility (hospital or health centre) attended by skilled attendant (Those who have trained to the level of Diploma and above) were categorized as “skilled care = 1”, and deliveries conduced at home or anywhere outside a health facility attended by un skilled attendant (family members, TBAs/TTBAs and HEWs) were categorized as ‘Unskilled care = 0’
6. Social support during labor	Having accompanying person during labor	Those who had someone (family member/relative) with them during the time of labor and delivery were labeled as ‘have social support = 1’ and otherwise ‘No social support = 0’
7. Immediate thermal care	Protecting the newborn from hypothermia by covering with clean cloth and placing in skin-to-skin contact with the mother	Neonates wrapped with clean cloth and placed in skin-to-skin contact with the mother were labeled as ‘appropriate thermal care = 1’ and otherwise ‘Not appropriate thermal care = 0’.
8. Breast-feeding initiation	The time the newborn first feed breast milk	Those started within 1 hour of birth were labeled as ‘timely initiated = 1 ’ and otherwise ‘delayed initiation = 0’
9. Clean and safe cord care	Using clean instrument to cut and tie the cord and putting nothing on the umbilical stump.	If cut by clean material, tied by clean thread and nothing is put on the stamp, it was labeled as “clean/safe cord care = 1’ if any one of these was missing, it was labeled as ‘Non-clean cord care = 0.’
10. Exclusive breast feeding of neonate	Giving only breast milk in the first 28 days	Thos who were on breast milk only were labeled as ‘on exclusive breast milk = 1’ and who started anything, including plain water were labeled as ‘Not on exclusive breast milk = 0’.
11. Newborn bathing	Washing the newborn for the first time	Those who were washed after the first 6 hours of birth were labeled as “appropriate bathing = 1’ & otherwise ‘not = 0’.
12. Vaccine on date of birth	Includes BCG and polio-0 on the date of birth	Neonates who received the 2 vaccines were labeled as ‘received the vaccines on date of birth = 1’ if one of them was missing, it was labeled as ‘no appropriate immunization on date of birth = 0’

**Table 2:** Description of variables and measurement for the study, Jimma Zone, Southwest Ethiopia, September 2012-April 2013

Level-2 predictor variables	Communal (kebele) characteristics	
Place of residence	The usual place of residence where the woman lives	Rural kebele was coded as ‘1’ and Urban kebele was coded as ‘2’.
Average distance from health centre	Approximate distance of respondent’s home from the nearest health centre on foot in munities as reported by respondent.	Average distance was computed for each kebele and dichotomized as ‘≤2hours = 1’ and ‘>2hours = 2’
Average distance from Hospital	Approximate distance of respondent’s home from the nearest hospital on foot in munities as reported by the respondent.	Average distance was computed for each kebele and categorized as ‘≤2 hours = 1’, ‘>2 hours =2’
Level-1 predictor variables	Individual and household characteristics	
Age	Age of women at interview in completed years	Categorized in to 7 groups by five-years interval, which later recoded in to three categories: ‘<20 = 1’, ‘20-29 = 2’ or ‘>29 = 3’
Ethnicity	The ethnic background of the respondent	Each ethnicity was entered and later recoded as ‘Oromo = 1’ and ‘Others = 2’. Others were merged because they were very few for logistic regressions.
Religion	The religious background of the respondent	Each religion was entered and later recoded as ‘Muslim = 1’ or ‘Others = 2’. Others were merged because they were very few for logistic regressions.
Educational status	Highest level of education attained by the respondent and her husband	Categorized in to 4 groups as ‘No Formal Education = 1’, ‘primary (1-8) = 2’, ‘Secondary or above (9^+^ = 3)
Occupational status	Current employment status and specific occupation of respondent and her husband	Categorized as ‘housewife’ (‘farmer’ for husbands) = 1, ‘employed = 2’, and ‘merchant = 3’
Wealth quintiles	Using EDHS questionnaire, house hold assets ownership were assessed and wealth index was computed by using principal component analysis	The wealth status was categorized in to five groups and ranked from poorest to wealthiest quintiles. ’First quintile =1’, ‘Second quintile = 2’, ‘Third quintile = 3, ‘Fourth quintile = 4’ and ‘Fifth quintile = 5’.
Birth order	Number of births a woman ever had including current birth	The responses was categorized in to three categories as: ‘1^st^ birth order =1’, ‘2^nd^ – 4^th^ = 2’ and ‘≥5^th^ birth order = 3’
Preceding birth interval	The duration between the current birth and the preceding birth in months.	The responses were categorized in to three as: ‘<24 months = 1’, ‘24-48 months = 2” and “>48 months = 3’.

### Data management and analysis

The collected data were coded and entered into Epidata V.3.1 to minimize logical errors and design skipping patterns. Then, the data were exported to SPSS for windows version 20.0 for cleaning, editing and analysis. Descriptive analysis was done by computing proportions and summary statistics. Wealth quintiles were determined by using Principal Component Analysis (PCA). Similarly, neonatal care practice, a continuous dependent variable, was created as a composite index (score) by using PCA.

The index was created by including the 12 elements of the minimum neonatal care package described in Tables 1 and 2 above. Each variable were measured in terms of "Yes" or "No" response categories and later changed to dummy variables by assigning "1" for "Yes" responses and "0" for "No" responses for the PCA. While doing the PCA, colinearities between the independent variables were checked by producing correlation matrix. However, no correlation coefficient was 0.9 or above for a variable to be excluded. The Kaiser-Meyer-Olkin (KMO) measure of sampling adequacy was 0.74 (>0.50 is acceptable) and Bartlett's Test of Sphericity was significant (*p* < 0.001). The importance of each variable for the model was checked by looking at the communalities and those variables having communalities < 0.5 were removed one by one from the model until all have ≥ 0.5. The eigenvalues was set to include values over 1.

Based on this, receiving social support during labor, receiving vaccination on date of birth and BPCR were excluded. As a result, 9 variables remained in the final model creating 4 principal components with eigenvalues > 1.0 explaining 65.04 % of the total variance (>60 % is acceptable to use PCA). No variable was found to have complex structure or high loadings (> 0.4 in more than one component in the rotated component matrix). Inter-item consistencies for the variables making each component were checked by Cronbach's alpha and all were > 0.7. The existence of outliers was checked by sorting each principal component by ascending order and all cases were within the range of ±3 factor scores. Finally, all the 4 components were added and an index (score), the continuous dependent variable, was created.

The status of neonatal care practice was determined by dichotomizing the score based on the mean value of the score. As Jimma town administration and Agaro town administration were included purposefully, weighted analysis was done to avoid urban over representation and over estimation of the status of neonatal care practices. The weighted analysis was done based on the complex-sample survey procedure by considering the probability of exclusion at different stages and the non-responses.

To identify factors affecting neonatal care practice, first, bivariate analysis was done to see associations between each independent variable and neonatal care practice. Then, all variables having *p* < 0.25 were considered as candidates for the multivariable model.

Because of the different levels of factors, a mixed-effects multilevel linear regression model was used by using STATA 13 to identify factors having significant associations with neonatal care practice. This model was preferred in order to avoid the clustering effects that violets the assumption of independence among the study subjects. To evaluate the existence of sufficient variance at the cluster level in influencing neonatal care practice, intercept-only model was fitted and Interclass Correlation Coefficient (ICC) was determined. Besides, goodness-of-fit of the multilevel model was tested by the log likelihood ratio (LR) test. Multicollinearity between the independent variables was assessed by using variance inflation factors (VIF >10 were considered as suggestive of existence of multicollinearity). In addition, cross-level two-way interactions were checked. Beta (β) coefficients along with 95 % CI were used to show the strength of the associations and level of significance.

The audio taped qualitative data were transcribed in to English language. Then, codes or terms were identified and tallied to come up with some categories, which later used to establish themes based on the objective of the study. Finally, thematic analysis was done and the findings were triangulated with the quantitative one.

### Ethical consideration

Ethical approval was obtained from the Institutional Review Board (IRB) of the College of Health Sciences of Addis Ababa University. In addition, written informed consent was obtained from each respondent before actual data collection.

## Results

### Socio-demographic characteristics

From the total of 3682 pregnant women enumerated and interviewed at the initial stage, 3612 pregnant women were included in the analysis at the baseline and enrolled to the follow up study after excluding 70 incomplete questionnaires. After excluding incomplete questionnaires, abortion cases, missed-to-follow up, maternal deaths and stillbirths, a total of 3463 live births happened and included in the analysis of this study making a response rate of 95.9 %. Of the total 3463 respondents, 75.1 % were from rural residence. Majority (63.8 %) of the mothers were in the age group of 20-29 years. Oromo was the dominant ethnic group (87.6 %) and Muslim was the leading religion (87.2 %). More than half (54.0 %), have not attended any formal education (Table 3).

**Table 3:** Socio-Demographic characteristics of Respondents, Jimma Zone, Southwest Ethiopia, September 2012-April 2013 (n = 3,463)

Variables	No.	Percent
Residence		
Urban	861	24.9
Rural	2602	75.1
Age (Years)		
<20	174	5.0
20-29	2209	63.8
≥30	1080	31.2
Ethnicity		
Oromo	3033	87.6
Amhara	169	4.9
Dawuro	96	2.8
Others*	165	4.7
Religion		
Musilim	3019	87.2
Orthodox	345	10.0
Protestant	99	2.9
Educational status		
No formal education	1871	54.0
Primary (1-8)	1270	36.7
Secondary (9-12)	256	7.4
>12	66	1.9
Occupation		
Housewife	3280	94.7
Employed (GO, NGO & Private)	78	2.2
Others^‡^	105	3.1
Husband’s Occupation		
Farmer	2459	71.0
Employed (GO, NGO & Private)	376	10.8
Merchant	413	11.9
Daily laborer	190	5.5
Others‡	25	0.8
Sex of neonates		
Male	1779	51.4
Female	1684	48.6
Types of birth		
Singleton	3387	97.8
Twins	76	2.2

*Yem, Kaficho, Guraghe and Tigrie, † Single, divorced and widowed, ^‡^Merchant, daily laborer and student

This table is Published with another objective on PLoS ONE 2014; 9(9): doi:10.1371/journal.pone.0107184.t002

### Neonatal care practices

Among the components of neonatal care practices during pregnancy, 53.8 %, 23.8 %, 41.9 % and 43.0 % received TT, planed for birth, received ANC and received adequate information on neonatal care, respectively. Among the elements of neonatal care during labor and delivery, 17.5 % and 95.0 % received skilled care and social support during labor, respectively.

Among the components after birth, 96.5 % received immediate thermal care, 64.1 % started breastfeeding within one hour of birth, 86.5 % got clean cord care, 91.5 % on exclusive breast-feeding, 56.5 % got bathing at appropriate time (after 6 hours of birth) and 8.1 % received vaccination (BCG and Polio-0) on the date of birth. By using these parameters, composite index was produced by using PCA and mean score was determined. Accordingly, 59.5 % (95 % CI: 57.6 %, 61.3 %) of neonates scored above or equal to the mean score and labeled as received good neonatal care (Table 4).

**Table 4:** Neonatal care practice in Jimma Zone, Southwest Ethiopia, September 2012-December 2013 (n = 3,463)

Variables	No.	Unweighted %	Weighted %
Received TT during pregnancy (at least 1 dose)	1962	56.7	53.8
Planed for birth and its complications	1202	34.7	23.8
Received skilled ANC Care at least once	1840	53.1	41.9
Adequate information on neonatal care	1501	43.3	43.0
Skilled care at birth	1064	30.7	17.5
Social support during labor and delivery	3268	94.4	95.0
Appropriate immediate thermal care	3359	97.0	96.5
Clean cord care	3042	87.8	86.5
Timely initiation of breast feeding (within 1 hour)	2307	66.6	64.1
Exclusive breast feeding (within 28 days)	3176	91.7	91.5
Appropriate bathing time (>6 hrs)	2160	62.4	56.5
Vaccines on date of birth (BCG & Polio 0)	425	12.3	8.1
Over all neonatal care practice			
Good Practice (≥Mean score)	2240	64.7	59.5
Poor Practice (<Mean score)	1223	35.3	40.5

The qualitative finding was also supplemented the quantitative one in that still there are problems in the coverage of some of the neonatal cares. The qualitative part particularly focused on breastfeeding, thermal care, cord are and neonatal immunization. According to the opinions of most of the respondents, previously there were problems in newborn feeding practices that most mothers do not start breast-feeding immediately. They also used to give fresh butter to the newborn to swallow with the assumption that he/she will not cry during the childhood. Now a days, the HEWs are educating the mothers and every mother gives breastfeeding immediately.

Concerning thermal care, mixed practices were reported by majority of the respondents. As repeatedly mentioned, during home delivery, almost all newborns are wrapped with clean new towel and put in front of mothers or someone caries them carefully. However, almost all mothers and newborns are washed just immediately or within thirty minutes by cold water. This is because of lack of knowledge about the importance of delayed bathing.

A 28 years old FGD discussant said, *"I gave birth to my child two months back by the help of traditional birth attendant. Just immediately, as the placenta was out, she washed me and my newborn with cold water. As to me, this is what all women in our community practice...."*

A key informant TBA added, *"…Both the mother and the newborn are contaminated with dirty blood. How can they stay with it for long hours? That is why we encourage immediate wash of both the mother and the newborn...."*

Concerning cord care, majority of the respondents had the view that previously, the mothers reuse rather blades to cut umbilical cord and put butter on umbilical stamp. But now, every woman knows its drawback and no such practices. Regarding to immunization, as reported by the majority, vaccination is the major problem of neonatal care. As most reported, previously mothers had no adequate knowledge and do not accept child immunizations. But now, every woman knows its benefits. However, the neonates are not getting appropriately because of many reasons from service providers. The major reported problems were unavailability of vaccines and when available, the providers do not open for few neonates.

A 24 years old FGD discussant stated, *"…I had taken my neonate two times to the health centre, but he never received the vaccine. On first day, the provider said, 'I can't open the vaccine for less than 10 children' and appointed me for a week. Again after a week, he said, 'no vaccine at all! Come another day!' I never went there again…"*

A HEW added, *"…we have been facing problems of open vial policy. We are not supposed to open BCG vial unless there are 10 neonates. We used to appoint mothers to bring on same day to open. But, they do not come on same day at same time. As a result, many neonates are not getting the BCG vaccine...."*

### Factors affecting neonatal care practice

To evaluate the applicability of the mixed-effects multilevel linear regression model, the ICC (ρ) was calculated in the empty-model and it was found to be 0.332 indicating that 33.2 % of the variation was contributed by between cluster variations. The test of the preference of log likelihood versus linear regression was also strongly significant (*P* < 0.0001). Then, the final full model was run by including all the cluster level and individual level variables and the ICC (ρ) became 0.157. This again indicated that 15.7 % of the variation was attributed to cluster level variables suggesting the preference of multilevel analysis. The preference of log likelihood versus linear regression was again strongly significant (*p* < 0.0001) (Table 5).

**Table 5:** Parameter coefficients and test of goodness-of-fit of the mixed effect multilevel model, in Jimma Zone, Southwest Ethiopia, September 2012-December 2013 (n = 3,463)

Models	Fixed intercept -cons (95 % CI)	Random effect as Level-2 variance		Intra-class Correlation Coefficient: ICC(ρ)	Log likelihood (LR) (deviance)	Significance of LR test Vs linear regression (*P*-value)
		var (-cons (95 % CI))	var (Residual (95 % CI))			
Empty model	0.25 (0.01, 0.49)	1.02 (0.72, 1.44)	2.05 (1.96, 2.15)	0.332 = 33.2%	−6269.60	<0.0001
Full model	−0.30 (-0.69, 0.09)	0.36 (0.25, 0.51)	1.94 (1.85, 2.04)	0.157 = 15.7%	−6143.08	<0.0001

After adjusting in the final two-level mixed-effects linear regression model, predictors of neonatal care practice existed both at the cluster level as well as at the individual level. Among the higher (cluster) level variables, place of residence was found to have statistically significant association with neonatal care practice. Being in urban residence was found to increase the neonatal care practice significantly (β =0.86; 95 % CI: 0.45, 1.23).

Among the lower level (individual) variables, maternal education, husband’s occupation, wealth quintiles, birth order and inter-birth interval were identified as predictors of neonatal care practice. Maternal education of primary (β =0.21; 95 % CI: 0.10, 0.32) and secondary or above (β = 0.76; 95 % CI: 0.55, 0.98) were found to increase the neonatal care practice significantly as compared with illiterate mothers. Mothers, whose husbands were employed (β = 0.54; 95 % CI: 0.30, 0.77) or merchants (β = 0.28; 95 % CI: 0.09, 0.47) had higher neonatal care practice as compared to those whose husbands were farmers.

Wealth quintiles of second (β = 0.18; 95 % CI: 0.03, 0.31), third (β = 0.30; 95 % CI: 0.15, 0.46), fourth (β = 0.41; 95 % CI: 0.25, 0.56) and fifth (β = 0.30; 95 % CI: 0.14, 0.46) also increased neonatal care practice significantly as compared to the lowest (poorest) wealth quintile. Similarly, inter-birth interval of 2-4 years (β = 0.20; 95 % CI: 0.01, 0.39) and above 4 years (β = 0.34; 95 % CI: 0.10, 0.58) increased neonatal care practice significantly as compared to interval of < 2 years. Birth order had inverse relationship with neonatal care practice. Birth order of 2^nd^-4^th^ (β = -0.30; 95 % CI: -0.52, -0.09) and above 4^th^ (β = -0.43; 95 % CI: -0.68, -0.19) significantly reduced neonatal care practice as compared to first-order neonates (Table 6).

**Table 6:** Multilevel analysis of factors affecting neonatal care practice, in Jimma Zone, Southwest Ethiopia, September 2012-December 2013 (n = 3,463)

Variables	Crude estimate β (95 % CI)	Adjusted estimate β (95 % CI)
Level-2 (higher level)-communal variables		
Place of residence		
Urban	1.63 (1.51, 1.75)	0.86 (0.45, 1.23)
Distance from health centre (on foot)		
>2hours	−0.58 (-0.70, -0.45)	−0.09 (-0.45, 0.27)
Distance from Hospital (on foot)		
>2hours	−1.38 (-1.54, -1.23)	−0.33 (-0.77, 0.11)
Level-1-lower level (individual) variables		
Age (Years)		
20-29	−0.55 (-0.82, -0.2)	−0.21 (-0.44, 0.03)
>29	−0.88 (-1.14, -0.59)	−0.25 (-0.52, 0.11)
Educational status		
Primary education (Grades 1-8)	0.52 (0.41, 0.64)	0.21 (0.10, 0.32)
Secondary or above (Grades ≥9)	1.95 (1.75, 2.14)	0.76 (0.55, 0.98)
Occupation		
Employed	1.20 (0.94, 1.45)	0.18 (-0.06, 0.41)
Husband’s occupation		
Employed	1.88 (1.71, 2,05)	0.54 (0.30, 0.77)
Merchant	1.28 (1.14, 1.42)	0.28 (0.09, 0.47)
Wealth index		
Second quintile	0.45 (0.27, 0.63)	0.18 (0.03, 0.31)
Third quintile	0.57 (0.38, 0.75)	0.30 (0.15, 0.46)
Fourth quintile	0.70 (0.52, 0.88)	0.41 (0.25, 0.56)
Fifth quintile	0.72 (0.54, 0.90)	0.30 (0.14, 0.46)
Birth order		
2^nd^ -4^th^	−0.58 (-0.73, -0.44)	−0.30 (-0.52, -0.09)
≥5^th^	−1.07 (-1.24, -0.91)	−0.43 (-0.68, -0.19)
Birth interval		
24-48 Months	0.34 (0.12, 0.56)	0.20 (0.01, 0.39)
>48 Months	0.93 (0.65, 1.21)	0.34 (0.10, 0.58)

The qualitative part also supplemented the quantitative one and the reasons for poor neonatal care were themed as low awareness, low-socio economy, costs and unavailability of transportations. Majority of the respondents had the feeling that most of the women, particularly in the rural areas, are illiterate and have no adequate knowledge about the risks of neonatal health problems and importance of neonatal care.

The other major problems repeatedly raised by most of the respondents as barriers to the neonatal care were unavailability of roads and means of transportation and costs of transportation and services to take them to health facility for preventive and curative services. The respondents also emphasized that majority of rural women are poor and give special emphasis to their daily work for family survival and give less attention to the neonatal care.

A 36 years old TBA expressed her sorrow as, *“…many women face problem because of road and transportation unavailability. The community has been trying to carry women after complication in labor. Now, they are helping by burying women because of repeated occurrences and loss of hope….”*

A 30 years old FGD discussant mother added, *‘….Lack of transport is our serious problem. I know one woman in my neighborhood. She had labor at home for more than a day. We tried to take her to health facility, but no any car around. As a result, we had no options except waiting her till the dead fetus came out ....”*

## Discussion

The overall status of neonatal care practice in this study was 59.5 %, which is relatively higher than previous study done in Addis Ababa (29 %) (Gashaw A. Assessment of New Born Care Practices During the first week of life Among mothers in Addis Ababa, Ethiopia (Unpublished)). However, majority of the components of the neonatal care practice along the continuum of care are still very low. For example, all the four components of neonatal care during pregnancy (receiving TT, planning for birth, attending ANC and receiving adequate information) were less than 50 %. Similarly, the coverage of skilled care at birth (conducted at health facility attended by skilled attendant) was very low (17.5 %). These findings are almost comparable with the national figures reported in EDHS 2011 [8]. This low coverage may be explained by the low awareness of mothers about neonatal health problems and the importance of neonatal care that might have lead to low utilization of the cares.

Care of umbilical cord always needs special attention as it can function as the entry point for infections. World Health Organization recommends dry cord care (where nothing is placed on cord stump unless indicated). Various studies done in developing countries have also reported that mothers apply substances like mustard oil, turmeric, cow dung and antiseptic lotion on the cord stump [21, 22]. In the contrary, clean cord care practice was found to be better (86.5 %) in this study. This may be due to difference in cultural practices in relation to neonatal care. In addition, the current community based interventions by health extension workers might have contributed to the reduction of traditional cord care practices in the study area.

The WHO recommends early initiation of breast milk (within one hour of birth) and avoiding extra feeding up to 6 months of age. In this study, 64.1 % of the newborn started breast milk within one hour of birth and 91.5 % were on exclusive breast milk during the first 28 days of birth. This is consistent with the finding of the study conducted in Addis Ababa in which 61.4 % of newborns started breast milk within the first hour of birth (Gashaw A. Assessment of New Born Care Practices During the first week of life Among mothers in Addis Ababa, Ethiopia (Unpublished)). Similar findings were also reported in studies done in Nigeria (65.3 %) [21] and Philippines (68.2 %) [22].

In this study, neonatal care practice was higher among mothers from urban residence as compared to the rural mothers. This is consistent with other prior studies [21]. This may be explained by the reason that women in urban areas have access to information and media and more likely to use ANC and skilled care at birth that might have contributed to this difference.

Similarly, neonatal care practice was found to increase significantly as educational status and wealth quintiles increase. Similarly, having a husband who is employed or merchant was found to increase neonatal care practice as compared to farmer husband. This is also consistent with other prior studies [22]. This may be explained by the reasons that when education increases, knowledge about health care, access to employment and income also increase. When there is high income or better wealth status, mothers are more likely to seek services for both themselves and their neonates. Having a husband, who is merchant directly mean that they have better income that enable the family to seek neonatal care.

In this study, first-birth-order neonates received significantly higher care as compared to higher-birth-order neonates. This may be due to the reason that families give special care for first child and this goes down as the number of live children increases sometimes because of negligence and economic issues to give care for all children. Inter-birth interval was another factor affecting neonatal care practice. Birth-interval of two years or above significantly increased neonatal care. This finding is similar with other studies conducted before [21]. The reason could be, when mothers get closely spaced births, they are expected to care for both children and themselves, which may lead to maternal exhaustion and negligence. As a result, the care for the later one gets decreased.

For policy and program implications, this study came up with the evidence that factors affecting neonatal care practice exist both at the community level as well as at the individual level. However, the community level predictor (place of residence) is more resistant to change. Therefore, interventions must focus on the individual level predictors, which are more feasible to be intervened. Moreover, as multiple factors exist at the individual level, multiple interventions need to be in place along the continuum of care encompassing all the elements of the minimum neonatal care package.

This study may have its own limitations in that all the findings concerning neonatal care practices were based on mothers own reports, which might have been affected by their memories and might introduced some biases, under reporting. The nature of the principal component analysis (PCA) includes the most important variables that contributed to the variation and excludes some other variables, which might have affected the status of neonatal care estimation, might have reduced the estimate. Therefore, it is important to consider these limitations while interpreting the findings of this study.

## Conclusions

This study indicated that the status of neonatal care practice in the study area was low. In addition, big variations were observed in the coverage of the components of neonatal care package. Particularly, the coverage of skilled care at birth and vaccination on the date of birth were very low. Factors affecting neonatal care existed both at the cluster as well as at the individual level. Place of residence, maternal education, husband’s occupation, wealth quintiles, birth order and inter-birth interval were identified as factors affecting neonatal care practice. Therefore, interventions targeting neonatal care should address all the components of the minimum neonatal care package along the continuum of care starting from before pregnancy, during pregnancy, during labor and after birth. Community level interventions need to be strengthened to address closely spaced births, improve ANC and skilled care use. Behaviour change communication (BCC) to the family, particularly mothers, also needs to be strengthened on the risks of neonatal health problems and the importance of neonatal care.

## Abbreviations

ANC: Antenatal Care
BPCR: Birth Preparedness and Complication Readiness
CI: Confidence Interval
FGD: Focus Group Discussion
GO: Governmental Organization
HEW: Health extension worker
IDI: In-depth Interview
MDG: Millennium Development Goal
NGO: Non Governmental Organization
PCA: Principal Component Analysis
TBA: Traditional Birth attendant
TT: Tetanus Toxoid
WHO: World Health Organization
